# Causal Diagrams: Pitfalls and Tips

**DOI:** 10.2188/jea.JE20190192

**Published:** 2020-04-05

**Authors:** Etsuji Suzuki, Tomohiro Shinozaki, Eiji Yamamoto

**Affiliations:** 1 Department of Epidemiology, Graduate School of Medicine, Dentistry and Pharmaceutical Sciences, Okayama University, Okayama, Japan; 2 Department of Information and Computer Technology, Faculty of Engineering, Tokyo University of Science, Tokyo, Japan; 3 Okayama University of Science, Okayama, Japan

**Keywords:** bias, causal inference, causality, confounding, directed acyclic graphs

## Abstract

Graphical models are useful tools in causal inference, and causal directed acyclic graphs (DAGs) are used extensively to determine the variables for which it is sufficient to control for confounding to estimate causal effects. We discuss the following ten pitfalls and tips that are easily overlooked when using DAGs: 1) Each node on DAGs corresponds to a random variable and not its realized values; 2) The presence or absence of arrows in DAGs corresponds to the presence or absence of individual causal effect in the population; 3) “Non-manipulable” variables and their arrows should be drawn with care; 4) It is preferable to draw DAGs for the total population, rather than for the exposed or unexposed groups; 5) DAGs are primarily useful to examine the presence of confounding in distribution in the notion of confounding in expectation; 6) Although DAGs provide qualitative differences of causal structures, they cannot describe details of how to adjust for confounding; 7) DAGs can be used to illustrate the consequences of matching and the appropriate handling of matched variables in cohort and case-control studies; 8) When explicitly accounting for temporal order in DAGs, it is necessary to use separate nodes for each timing; 9) In certain cases, DAGs with signed edges can be used in drawing conclusions about the direction of bias; and 10) DAGs can be (and should be) used to describe not only confounding bias but also other forms of bias. We also discuss recent developments of graphical models and their future directions.

## 1. BACKGROUND ON THE TOPIC

Causal diagrams have been often used among epidemiologists as a tool to describe what is already known about relevant causal structures. In 1999, Greenland et al introduced formal theories of causal directed acyclic graphs (DAGs) within epidemiology,^1^ and several comprehensive introductions to DAGs have been published,^2^^–^^7^ including a review article in Japanese.^8^ Consequently, the use of DAGs is now widespread among epidemiologists; when consulting *A Dictionary of Epidemiology*, although there was no entry for DAGs in its fourth edition that was published in 2001,^9^ a definition of DAGs has been included in its later editions.^10^^,^^11^

Confounding is one of the primary concerns in epidemiologic research, and DAGs are used extensively to determine the variables for which it is sufficient to control for confounding to estimate causal effects. A confounder was traditionally identified based on the following three criteria^3^^–^^5^: a) it must be associated with the exposure; b) it must be associated with the outcome in the unexposed; and c) it must not lie on a causal pathway between exposure and outcome. Because these traditional criteria sometimes fail, however, the graphical criteria for identifying confounders in DAGs are especially useful. This point is often explained using an example of the so-called M bias.^12^ In this case, even if one takes care to not adjust for variables affected by exposure or outcome in the traditional confounder-selection criteria, one may be led to adjust for a “collider” on the backdoor path from exposure to outcome and unnecessarily introduce bias.

Despite its widespread use, however, the lack of clear understanding of this tool could lead to inappropriate use and underappreciation of DAGs. The goal of this paper is not to provide a basic introduction of DAGs; rather, we aim to review some pitfalls that are easily overlooked when using DAGs and discuss tips, hoping that DAGs will be further used in epidemiology as well as in a wide range of other research disciplines. We also discuss recent developments of graphical models and their future directions.

## 2. PITFALLS AND TIPS

Simply speaking, a DAG is composed of nodes (or vertices) and arrows (or arcs/edges) connecting them such that the graph is acyclic. The first three pitfalls below address these basic components, followed by other pitfalls when drawing and interpreting DAGs.

### 2-1. Each node on DAGs corresponds to a random variable and not its realized values

When drawing DAGs, we are primarily concerned about causal relations among variables. DAGs are a simple way to encode our subject-matter knowledge, or our assumptions, about the qualitative causal structure of interest. Consequently, each node on DAGs corresponds to a random variable and not its realized values. Incorrect use of nodes can lead to erroneous construction of the DAGs. For example, Steiner et al proposed that propensity score itself is described as a collider with respect to exposure and confounders, claiming that propensity score analysis removes the confounding bias by offsetting the relation from confounders to exposure via association due to conditioning on the collider, regardless of analytic method (eg, matching, stratification, or weighting).^13^ Their approach, however, does not describe causal structures between random variables; rather, they are concerned about the realized values of propensity scores based on an empirical joint distribution of exposure *A* and confounders *C* in a sample. Also, the fact that (true) propensity score is the functional of the joint probability distribution *P*(*A*, *C*) does not justify drawing arrows from *A* and *C* to the node representing propensity score. Arrows in DAGs should represent causal structure between each random variable but not the dependence forms defined by probability distributions; a related pitfall is presented in Section 2-2.

Note that statistical independencies implied by a DAG are only “in expectation”, and they apply to the expected data distribution if the causal structure represented by the DAG is correct. Thus, the associations that may arise as a result of purely random events, such as those produced by randomization or random sampling, are not described in DAGs. A related issue is discussed in Section 2-5.

### 2-2. The presence or absence of arrows in DAGs corresponds to the presence or absence of individual causal effect in the population

We say that *P* affects *Q* in a population if and only if there is at least one individual for whom changing (intervening on) *P* would change *Q*.^4^ In line with this, an arrow from *P* to *Q* is drawn when we suspect there is a direct causal effect (ie, an effect not mediated through any other variables in the DAG) for at least one individual in the population, or when we are unwilling to assume such individual causal effects do not exist.^4^^,^^5^ Thus, the presence of an arrow from *P* to *Q* does not necessarily imply that these two variables are statistically dependent; if the quantity of positive individual causal effect and that of negative individual causal effect are equivalent, they perfectly cancel out and the net effect of *P* on *Q* becomes null (this situation would be relatively rare in large samples). If this is the case, *P* and *Q* are statistically independent even if there is an arrow from *P* to *Q*. By contrast, if there are no (direct or indirect) paths from *P* to *Q*, it means that we assume that a sharp null hypothesis of no causal effect of *P* on any individual’s *Q* holds in the population.^4^^,^^5^ Thus, omitting arrows represents a strong assumption, though some may put more emphasis on drawing arrows. Recall that the mathematical theory underlying the graphical rules is known not as “d-connection” but as “d-separation”.^2^ To summarize, the presence or absence of arrows in DAGs corresponds to the presence or absence of individual causal effect in the population.

**Figure 1.**
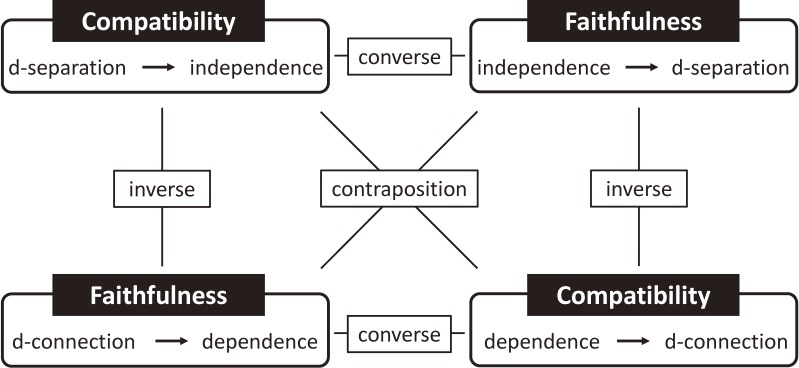
The relation between compatibility and faithfulness. See Section 2-2 and Box 1 for details.

This point is sometimes explained using the rules of *compatibility* and *weak faithfulness* (see Figure 1 and Box 1 for their definitions).^3^ Under these two rules, we can specify the relation between d-separation and statistical independence, and it is consistent with drawing arrows based on the presence or absence of individual causal effect. When we further assume *faithfulness* (see Figure 1 and Box 1 for its definition), we can also find the statistical dependencies implied by DAGs.^3^ (Matching leads to unfaithfulness: see Section 2-7 and elsewhere for details.^14^^,^^15^) We may often see apparent violations of faithfulness in small data sets when that would not be the case in a larger sample from the same population. However, because faithfulness is controversial as a routine assumption from practical as well as theoretical perspectives, Glymour and Greenland discuss only uses of graphical models that do not rely on its assumption in *Modern Epidemiology*,^3^ whereas Hernán and Robins assume faithfulness throughout their book *Causal Inference: What If* unless mentioned otherwise.^5^ The importance of faithfulness becomes clearer for “causal discovery”. The details about these rules are beyond the scope of this paper, but it is notable that, under the rule of compatibility and the assumption of faithfulness (which are collectively referred to as *perfect compatibility*),^3^ the presence or absence of arrows corresponds to the presence or absence of average causal effect in the target population, instead of individual causal effect. These may well illustrate the significance of distinguishing individual causal effect and average causal effect.

**Box 1.** Terminology in Section 2-2

•	**d-separation/d-connection:** Two variables (or two sets of variables) are said to be d-separated if every path between them is blocked; otherwise they are d-connected.
•	**Rule of *compatibility*:** Whenever two sets of variables are d-separated given a third set, the two sets are independent conditional on the third.
•	**Assumption of *faithfulness*:** Whenever two sets of variables are d-connected given a third set, the two sets are associated conditional on the third. This is the converse property of compatibility.
•	**Rule of *weak faithfulness*:** When two sets of variables are d-connected given a third set, the two sets are expected to be associated conditional on the third.

In the Appendix, a further discussion is provided following an example in Fine Point 6.2 of *Causal Inference: What If*.^5^

### 2-3. “Non-manipulable” variables and their arrows should be drawn with care

In the counterfactual framework, we think of the effect of a particular factor as a contrast between the potential outcomes when an exposure is set to a particular value, while holding all other components constant (unless they are assumed to mediate the causal path).^16^^,^^17^ Therefore, if we can clearly address hypothetical interventions on manipulable variables, it is relatively straightforward to examine their causal effects. Because a counterfactual definition of causation requires hypothetical intervention(s), the question of whether non-manipulable variables can be considered “causal” has long been controversial.^4^ For example, Holland famously argued that “causes are only those things that could, in principle, be treatments in experiments”, putting forward the slogan “no causation without manipulation”.^18^ Although counterfactuals related to manipulable quantities are of great value, causation related to non-manipulable quantities can be of scientific interest and constitute a substantial portion of instances of causation in biomedical and social sciences.^19^ For example, though not easily amenable to experimental interventions, we may be interested in examining “causal” effects of sex, race, or genetic ancestry. There have been similar arguments about age-period-cohort analyses; although this analytic method is used to measure the effect of time, hypothetical interventions to change “time” are beyond the realm of possibility, though may be conceivable.^20^

Accordingly, when drawing DAGs, each researcher should carefully think about whether it even makes sense to include non-manipulable variables at all with any arrows into or out of them. In this regard, it would be helpful to clearly define “hypothetical interventions” in the counterfactual framework.^21^ If a “hypothetical intervention” on a non-manipulable variable is well-defined, the variable may be treated as a “hypothetically manipulable” variable. For particular research hypotheses of interest, we may need to represent “hypothetically manipulable” variables (either as exposures or as covariates) and show arrows emanating from such variables. In most cases, however, “hypothetically manipulable” variables will not have any causes (other than the hypothetical intervention) and therefore will not have arrows pointing into them.^4^ Consequently, for example, the total causal effect of sex can be estimated simply by taking the observed differences in the outcome of interest between male and female.^19^ In other words, once we adopt the presence of “causal” effect of sex on a particular health outcome, there is no confounding because sex itself is randomized at conception. As one of a handful of exceptions, Glymour explained that, if sex ratios of newborns are thought to vary slightly in response to environmental conditions,^22^ it may be useful to include such stressors as causes of sex.^4^ To summarize, “non-manipulable” variables and their arrows should be drawn with care from a perspective of hypothetical interventions.

On a related issue, even if the exposure of interest is manipulable and hypothetical interventions on it are conceivable, there is a serious concern arising from ill-defined causal questions. If there are multiple versions of treatment, which is referred to as compound treatments,^5^^,^^23^ estimating causal effects is challenging in randomized controlled trials (RCTs) as well as in observational studies. See Hernán and VanderWeele for DAGs with compound treatments.^23^

### 2-4. It is preferable to draw DAGs for the total population, rather than for the exposed or unexposed groups

The target population concept plays a key role in discussions of causal inference in epidemiology, and it has been well established that confounding depends on the population chosen as the target of inference.^24^^–^^26^ The target can be the total population or the exposed, the unexposed, or conditional on the covariates. When drawing DAGs, however, it would be preferable to use the total population as the target population, which enables us to more readily identify the presence of bias.^26^^,^^27^ This is because, as is further explained in Section 2-5, DAGs are primarily useful to examine the presence of confounding in distribution in the notion of confounding in expectation.^26^ The exposed and unexposed groups are, by definition, determined by the specific pattern of exposure status. Thus, if these groups are used as target populations, the discussion is logically restricted to the notion of realized confounding for the two target populations in that particular pattern. Generally, drawing DAGs for the exposed or unexposed group should be avoided.^27^^,^^28^

If we intend to use the exposed and unexposed groups as target populations when drawing DAGs, we may need to draw a square around the node of exposure to explicitly indicate that we are conditioning on exposure status. On a related issue, other than unspecified component causes, when every sufficient cause either includes both the exposure and a certain covariate (ie, causal co-action), or includes the exposure alone without the covariate, confounding does not occur if the target population is the exposed group. This is because, irrespective of distributions of the covariate and the unspecified component causes, no outcome occurs in the actual unexposed group, which is used as a substitute of what would have occurred in the actual exposed group had they been unexposed. In this situation, Flanders et al proposed a simple modification of DAGs to use dotted arrows for all effects that are absent when exposure is absent.^29^ Their approach, however, is also based on DAGs for the total population.

### 2-5. DAGs are primarily useful to examine the presence of confounding in distribution in the notion of confounding in expectation

Despite its significance, the different notions of confounding have not been fully appreciated in the literature, leading to confusion of causal concepts in epidemiology. This lack of clear understanding could lead to inappropriate use and underappreciation of DAGs, which provide a simple algorithm for examining the presence of confounding in distribution in the notion of confounding in expectation.^26^ We briefly explain these notions of confounding below.

First, the notion of confounding can be defined both with respect to marginal distributions of potential outcomes (ie, *confounding in distribution*) and with respect to a specific effect measure (ie, *confounding in measure*).^30^^,^^31^ By definition, confounding in distribution is scale-independent, whereas confounding in measure is scale-dependent. No confounding in distribution is a sufficient condition for no confounding in measure. DAGs are primarily useful to examine the presence of confounding in distribution because they are completely nonparametric and provide qualitative assumptions behind a causal analysis.^1^^,^^26^ As explained in Section 2-4, it is preferable to draw DAGs for total population as the target population. When the target is the total population, confounding depends on the notions of confounding in distribution and confounding in measure,^26^^,^^30^^–^^32^ which highlights the significance of distinguish these notions.

Second, a further distinction can be drawn between *confounding in expectation* and *realized confounding*.^26^^,^^30^^,^^33^ In an ideal RCT, the randomized groups will be comparable in their potential outcomes on average over repeated experiments. For any given experiment, however, the particular randomization may result in imbalances by chance because of the particular allocation or exposure assignment.^34^ Such a scenario would be one in which there is no confounding in expectation but there is realized confounding for the particular experiment.^26^^,^^30^ (This phenomenon has been also referred to as random confounding,^35^ and is particularly of concern when the size of the population is small.) To grasp the profound distinction between these notions of confounding, we need to understand the mechanism that generates exposure events not the product of that mechanism. The lack of clear understanding of this mechanism could lead to inappropriate use of DAGs to examine the presence of realized confounding.^27^^,^^28^ DAGs are, however, primarily useful to examine the presence of confounding in expectation,^26^ which is a form of systematic error.^36^ Therefore, one needs to draw DAGs based on the understanding of underlying causal structures among random variables, as mentioned in Section 2-1. This point is related to the fact that bias (or more strictly speaking, exact bias^5^) is defined by comparing the expected value of an estimator and the true value of the parameter.^37^^,^^38^ If the size of population is large enough, DAGs are also practical tools to identify realized confounding.^26^

To summarize, DAGs are primarily useful to examine the presence of confounding in distribution in the notion of confounding in expectation.^26^ See elsewhere for a detailed discussion about the notions of confounding.^26^

### 2-6. Although DAGs provide qualitative differences of causal structures, they cannot describe details of how to adjust for confounding

Analytic adjustment for confounders in observational studies has consequences quite different from those of physical control in RCTs. When randomization is implemented in ideal RCTs, there is no confounding in expectation, and all arrows pointing into exposure(s) can be erased or removed.^26^ In observational studies, however, arrows pointing into exposure(s) cannot be erased, even though one aims to adjust for confounding by using appropriate study designs as well as analytic methods. There are inherent distinctions in the underlying causal structures between observational studies and RCTs, which can be readily understood by considering theoretical data frequencies in these studies based on the counterfactual model.^39^

Although DAGs can provide qualitative differences of causal structures between observational studies and RCTs, they cannot describe details of how to adjust for confounding because DAGs are not quantitative models. For example, we often use a square box around a node to indicate that we are conditioning on it (or stratifying on its values). If we can identify a sufficient set of covariates to block all the backdoor paths from exposure to outcome, the statistical relation between exposure and outcome *within strata of the covariates* is due only to the effect of exposure on outcome *within the strata*. Indeed, blocking the flow of association between exposure and outcome through the common cause(s) is the graph-based justification to use stratification as a method to achieve exchangeability.^5^ However, we cannot use DAGs to explain the fact that standardization can be used to obtain marginal causal effect in the target population; rather we need to analytically explain that marginal causal effect becomes equivalent to a weighted average of conditional causal effects. Thus, DAGs cannot be used to distinguish analytic methods that aim to obtain conditional causal effects (eg, stratified analyses and regression models) and those that aim to obtain marginal causal effects (eg, standardization). Further, DAGs cannot be used to explain whether specific estimators or estimation works well. Irrespective of the methods employed to adjust for confounding, however, DAGs can be used to determine the variables for which it is sufficient to control for confounding to estimate causal effects.

### 2-7. DAGs can be used to illustrate the consequences of matching and the appropriate handling of matched variables in cohort and case-control studies

The goal of matching differs by study design. In cohort studies, matching is used to prevent confounding by the matched variables, and then adjustment for these variables may be unnecessary to remove bias in point estimation. In case-control studies, matching is used to increase statistical efficiency. Unlike cohort matching, case-control matching does not and cannot remove confounding, but instead may induce selection bias that can itself be removed by adjustment for the matched variables. Although these differences were clarified long ago, misconceptions about the implications of matching remain common. DAGs can be used to illustrate the consequences of matching and the appropriate handling of matched variables in cohort and case-control studies.^14^^,^^15^ Let *A* denote an exposure, *Y* denote an outcome, and *C* denote a confounder and matching variable. The variable *S* indicates whether an individual in the source population is selected for the matched study (1: selected, 0: not selected).

**Figure 2.**
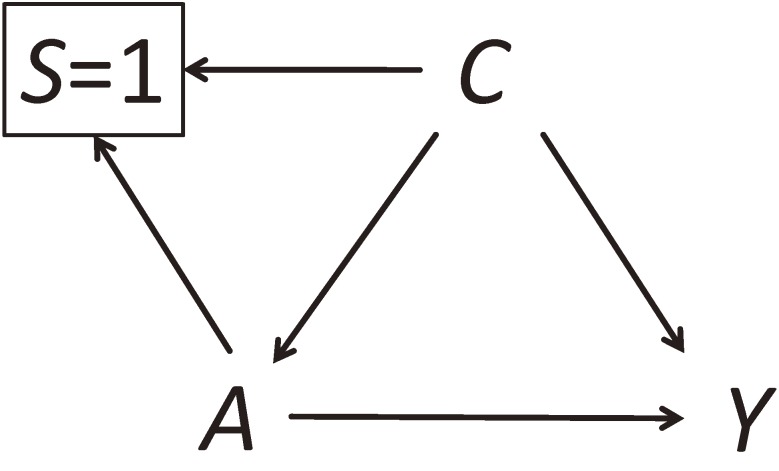
Cohort matching on a confounder. We let *A* denote an exposure, *Y* denote an outcome, and *C* denote a confounder and matching variable. The variable *S* indicates whether an individual in the source population is selected for the matched study (1: selected, 0: not selected). See Section 2-7 for details.

In cohort studies, matching process ensures that the distributions of matched variables are (nearly) identical across exposure groups. In a DAG, cohort matching is described by drawing arrows from *C* and *A* to *S* because selection into the sub-cohort depends on the values of *C* and *A* (Figure 2). The square around *S* = 1 indicates that the analysis is conditional on having been selected. Although the variables *C* and *A* are d-connected via two paths, *C*→*A* and *C*→$\begin{array}{c} S=1 \end{array}$←*A*, these variables are independent by design. In other words, the two paths “unfaithfully” cancel each other exactly. This graphical representation is useful to explain that, if there are unmatched confounders in matched cohort studies, ignoring the matching variables may leave bias even when additional adjustment is made for the unmatched confounders.^40^ A similar issue occurs for propensity score balancing.^41^

**Figure 3.**
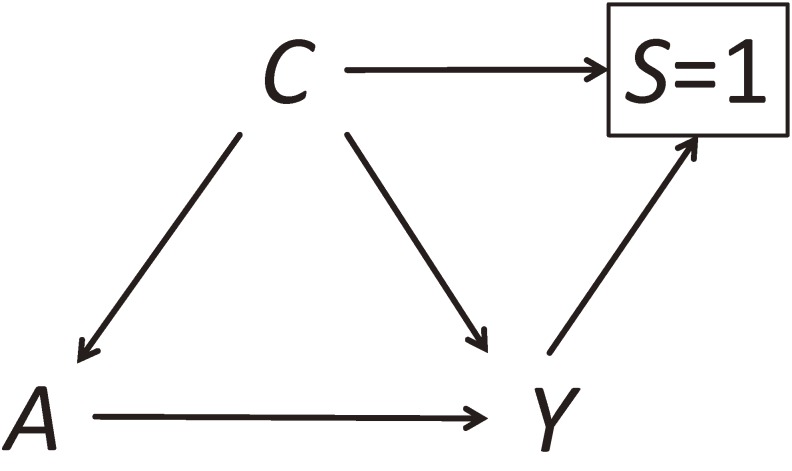
Case-control matching on a confounder. We let *A* denote an exposure, *Y* denote an outcome, and *C* denote a confounder and matching variable. The variable *S* indicates whether an individual in the source population is selected for the matched study (1: selected, 0: not selected). See Section 2-7 for details.

By contrast, case-control matching ensures that the distributions of matched variables are (nearly) identical across outcome groups. In a DAG, case-control matching is described by drawing arrows from *C* and *Y* to *S* because, by definition, both *C* and *Y* affect *S* (Figure 3). (Here, we only discuss a situation in which there is an arrow from *A* to *Y*. See elsewhere for a situation without the arrow.^14^^,^^15^) The variables *C* and *Y* are d-connected via three paths, *C*→*Y*, *C*→*A*→*Y*, and *C*→$\begin{array}{c} S=1 \end{array}$←*Y*. The third path is induced by the matching and is of equal magnitude but opposite direction to the net association via the first two paths. Consequently, *C* and *Y* are independent unconditional on *A* in the matched sample (ie, unfaithfulness). This implies, however, that the net association over the two paths *C*→*Y* and *C*→$\begin{array}{c} S=1 \end{array}$←*Y* is not zero: *C* and *Y* are associated conditional on *A* in the matched sample. Therefore, the case-control matching does not break the biasing path *A*←*C*→*Y*, which graphically explains that case-control matching does not prevent the original confounding. Moreover, conditional on *S* = 1, there is a biasing path for the effect of *A* on *Y*, *A*←*C*→$\begin{array}{c} S=1 \end{array}$←*Y*, which graphically explains that case-control matching induces selection bias. Adjustment for *C* is necessary to control both the intentional selection bias introduced by matching and the original confounding. Furthermore, case-control matching on a non-confounder may lead to selection bias, which is illustrated in DAGs.^3^^,^^14^^,^^15^ For details about DAGs for matching, see elsewhere.^14^^,^^15^

Finally, it is worth mentioning that, when using a square box around a node, there are two distinct ways. One is to simply add a square around a node, which is often used to indicate that all the strata of the variable are observed (eg, when conditioning is done analytically). The other is to use a square around a node by specifying the value on which conditioning was made (as in Figure 2 and Figure 3). This is also often employed to indicate that the strata except for the one indicated are missing (eg, when conditioning is done during selection or attrition, which could result in selection bias). Because the values on which conditioning was made could yield different associations, it is important to specify them when drawing DAGs.^42^

### 2-8. When explicitly accounting for temporal order in DAGs, it is necessary to use separate nodes for each timing

DAGs are, by definition, acyclic, and they should contain no feedback loops. Therefore, no variable is an ancestor or descendant of itself. If *A* causes *Y*, *Y* cannot also cause *A* at the same time. Though it may induce few problems in settings of a single-point exposure, we need to carefully draw DAGs for longitudinal analyses with time varying exposures by using separate nodes for each timing. If a prior value of *Y* affects *A*, and then *A* affects a subsequent value of *Y*, we must use separate nodes because these are separate variables (eg, *Y*_0_ → *A*_1_ → *Y*_2_).

On a related issue, some researchers draw DAGs without a rigorous consideration of chronological order, putting more emphasis on their visibility. Others are more rigorously concerned about the chronological order, consistently drawing variables that occur temporarily earlier on the left side of DAGs.^5^ Furthermore, some may draw DAGs in the order from top to bottom,^43^ and others may draw in the order from right to left. These are merely differences of drawing styles of DAGs, and there is probably no single correct style. To avoid pitfalls in drawing DAGs, however, it may be helpful to prioritize clarity of communication. For example, although M bias is a classic example to illustrate benefits of using DAGs,^12^ its form becomes quite different if chronological orders of variables are rigorously drawn. It is thus perhaps helpful to use different styles properly based on the aims of using DAGs.

### 2-9. In certain cases, DAGs with signed edges can be used in drawing conclusions about the direction of bias

Arrows in DAGs represent the presence of causal relations between the variables, and do not describe their directions or signs. Consequently, DAGs cannot describe direction of bias. This point is crucial because when interpreting epidemiologic findings, it is significant to consider not only the presence of but also the direction of bias—whether underestimation or overestimation occurs.

To address this issue within the context of DAGs, signs can sometimes be added to the edges of DAGs following the rigorous rules.^44^^–^^46^ Briefly, a positive sign can be added to an edge from *A* to *Y* if a distributional positive monotonic effect of *A* on *Y* is met, which means that intervening on *A* will increase or leave unchanged the distribution of *Y* over the population, regardless of the value of the parents of *Y* other than *A*.^45^ A negative sign can be analogously added. If *A* has neither a distributional positive monotonic effect nor a distributional negative monotonic effect on *Y*, then the edge from *A* to *Y* is said to be without a sign. The sign of a path on a DAG is the product of the signs of the edges that constitute that path. If one of the edges on a path is without a sign, then the sign of the path is said to be undefined. Based on these rules, DAGs with signed edges, or signed DAGs, can be used to draw conclusions about the direction of the bias of unmeasured confounding under specific assumptions. Weaker assumptions can be drawn in certain situations.^44^

When using signed DAGs, the direction of nodes themselves should be also clearly indicated; for example, instead of a node for “sex”, we need to use a node for “male”.

### 2-10. DAGs can be (and should be) used to describe not only confounding bias but also other forms of bias

Because DAGs have been used extensively to determine the variables for which it is sufficient to control for confounding to estimate causal effects, some may misunderstand that DAGs can be used only for assessment of confounding bias. To obtain an accurate estimate of the causal effect of exposure on outcome, however, we need to deal with other important forms of bias.^36^ If DAGs are used only as tools to assess confounding bias, we may overlook other important bias and fail to properly assess them.

DAGs can be used to illustrate not only confounding bias but also selection bias and measurement bias.^42^^,^^46^^,^^47^ It is fairly well known that both confounding bias and selection bias imply a lack of exchangeability between the exposed and unexposed groups,^33^^,^^39^^,^^42^^,^^48^ and they may be collectively referred to as nonexchangeability bias.^36^ In some cases, confounding bias and selection bias cannot be clearly distinguished, and the same is true in DAGs. The blurred border between confounding bias and selection bias can be illustrated using M bias^42^; while some prefer to classify M bias as selection bias because it is induced by conditioning on a common effect of causes of exposure and outcome,^42^ others may prefer to refer to it as confounding bias based on the position of the entire path in the DAG.^3^ Even if the distinction between confounding bias and selection bias cannot be clearly made, DAGs can enhance communication among investigators to avoid unnecessary misunderstandings. Thus, even though analyses may be conditioned on participants in a study or survival to a certain age, it is preferable to include that selection process in the DAG, rather than omit it entirely. Unlike confounding bias and selection bias, measurement bias is explained by the presence of backdoor path(s) between the misclassified exposure and the misclassified outcome.^36^ DAGs can be used to represent four types of measurement error: independent nondifferential, dependent nondifferential, independent differential, and dependent differential errors.^46^^,^^47^

To summarize, DAGs can be used to illustrate these three main source of bias (ie, confounding bias, selection bias, and measurement bias); a recent paper refers to these biases as structural error.^36^ DAGs can be used at various steps during the process of conducting research, contributing to understanding of the overall picture of errors in causal inference. Even in RCTs, because randomization at baseline does not guarantee exchangeability during the post-baseline period (eg, due to differential loss to follow-up), it is significant to consider the whole process of obtaining data when drawing DAGs. For details of illustration, see elsewhere.^49^^,^^50^

## 3. FUTURE DIRECTIONS

Graphical models are useful tools in causal inference. In this paper, we have primarily focused on causal DAGs, which provide one basis of our clearer understanding of causality if appropriately used. In addition, there are other important causal models aside from graphical ones. Greenland and Brumback discussed four major causal model frameworks^51^; graphical models, potential outcome (counterfactual) models, sufficient cause models, and structural equation models. These frameworks are distinct but closely related, providing complementary perspectives of causality. For example, DAGs are often criticized for not allowing for the representation of interactions among variables. In this regard, VanderWeele and Robins proposed to incorporate sufficient causes into the DAG framework, in which an ellipse is put around the sufficient-cause nodes to indicate that the set is determinative.^52^^,^^53^ Causal DAGs with sufficient causation structures thus allow for the graphical representation of interactions on DAGs. Furthermore, by formulating mediation in the sufficient cause framework,^54^^,^^55^ Suzuki et al showed that operating mediation and mechanism can be identified from empirical data, illustrating it in causal DAGs with sufficient causation structures.^56^ A similar approach was used to help distinguish and relate concepts of confounding and of covariate balance.^57^ These approaches provide the link between the graphical models and the sufficient cause models among the four major causal model frameworks. Further, DAGs represent nonparametric structural equation models,^2^^,^^5^ and the link between graphical models and potential outcome models becomes clearer using advanced types of causal diagrams, as described later. In these manners, attempts to integrate the four major causal model frameworks can lead to profound, universal perspectives of causality.^58^^–^^62^ Further developments of causal theories would enhance versatility of causal diagrams in applied research. Meanwhile, the significance of study design itself should not be overlooked for valid causal inference, and it is important to have a clear research question before even beginning to draw a DAG.^8^

Although DAGs are useful tools to make decisions for confounder selection, one can rarely construct perfectly correct DAGs because complete knowledge of underlying causal structures is often unavailable. Uncertainties prevail in biomedical and social sciences.^63^ Accordingly, VanderWeele proposed a practical approach to confounder selection decisions^64^: control for each covariate that is a cause of the exposure, or of the outcome, or of both; exclude from this set any variable known to be an instrumental variable; and include as a covariate any proxy for an unmeasured variable that is a common cause of both the exposure and the outcome. This approach is referred to as a “modified disjunctive cause criterion”, which would be a useful guide for confounder selection, once we can properly use DAGs to avoid the pitfalls.

Finally, conventional causal diagrams do not include the underlying counterfactual variables on the graphs. In other words, the link between the graphical models and the potential outcome models has remained traditionally hidden.^5^ In this regard, Richardson and Robins developed single world intervention graphs (SWIGs),^65^ which explicitly connect the potential outcome framework with DAGs. Although DAGs are used to visually summarize hypothetical relations among observed variables, SWIGs allow us to show hypothetical relations between observed/unobserved factual random variables and potential (or counterfactual) outcomes. This advanced type of causal diagram is introduced primarily in a chapter about confounding of *Causal Inference: What If*,^5^ and its further use is expected. Although SWIGs have been used less in other settings, a recent letter discussed a practical example demonstrating the utility of SWIGs for selection bias.^66^ As another approach to describe hidden causal structures, Suzuki et al proposed extended DAGs, which integrate response types and observed variables.^39^ They show the conceptual link between unobservable response types and observed (or observable) data frequencies in the population. This is crucial because the causal effect of exposure on disease frequency in a population depends on the distribution of response types of individuals in that population. As an example of their usefulness, the principal stratification approach can be illustrated using extended DAGs to describe such contexts as truncation by death, mediation, and noncompliance.^67^ Some other extensions of DAGs include selection diagrams^68^ and missingness graphs.^69^

As addressed in this article, there are many pitfalls to avoid when using DAGs. Indeed, DAGs are not panacea, and there is no magic bullet for causal inference. Nevertheless, DAGs have proven to be a useful tool to clarify our causal thinking. As a visual aid, causal diagrams provide an incomparable help at the stages of study design, data collection and analysis, and interpretation of study findings. Furthermore, their value as a pedagogical tool cannot be overlooked in the causal inference literature. It is hoped that the utility of DAGs will be further advanced as an interdisciplinary tool, and thus contribute to elaborate scientific understanding of causality.

## Figures and Tables

**Figure 4.**
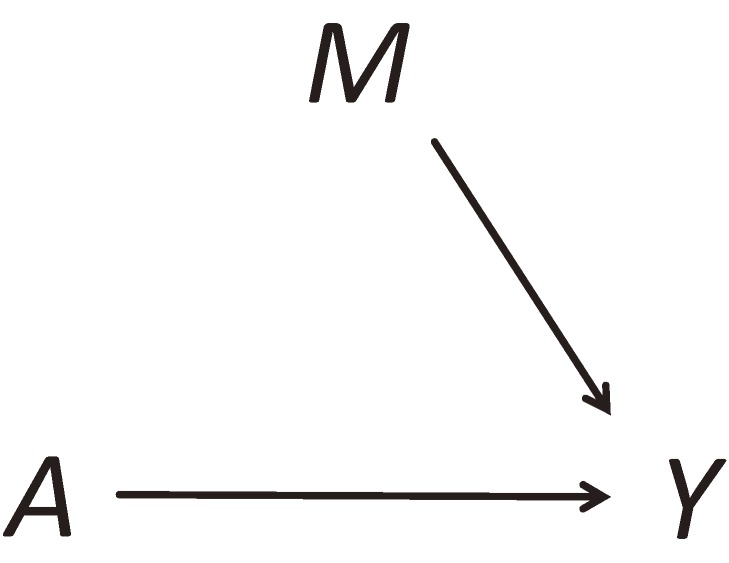
DAG that properly illustrate the underlying causal structures, before conditioning on *M*. We let *A* denote a binary exposure (1 = received heart transplant, 0 = did not receive heart transplant) and *Y* denote a binary outcome (1 = deceased, 0 = did not decease) and *M* denote a binary covariate (1 = men, 0 = women). DAG, directed acyclic graph.

**Figure 5.**
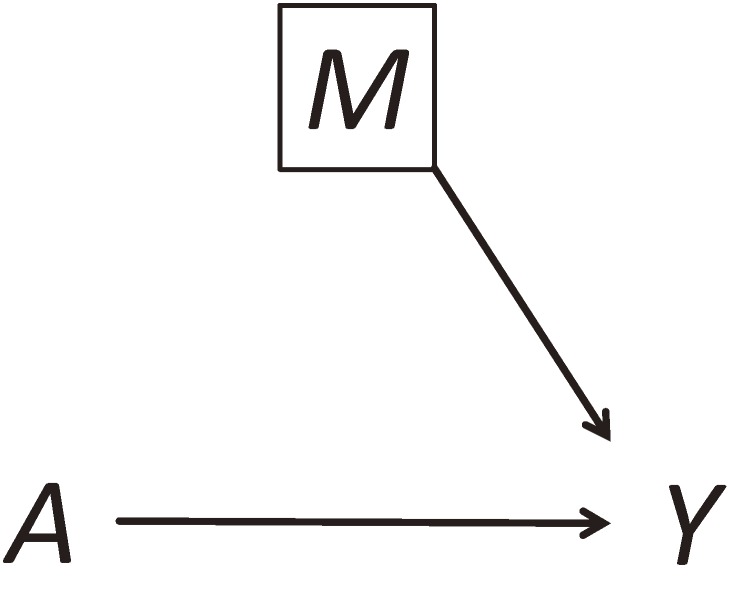
DAG that properly illustrate the underlying causal structures, after conditioning on *M*. We let *A* denote a binary exposure (1 = received heart transplant, 0 = did not receive heart transplant) and *Y* denote a binary outcome (1 = deceased, 0 = did not decease) and *M* denote a binary covariate (1 = men, 0 = women). DAG, directed acyclic graph.

**Figure 6.**
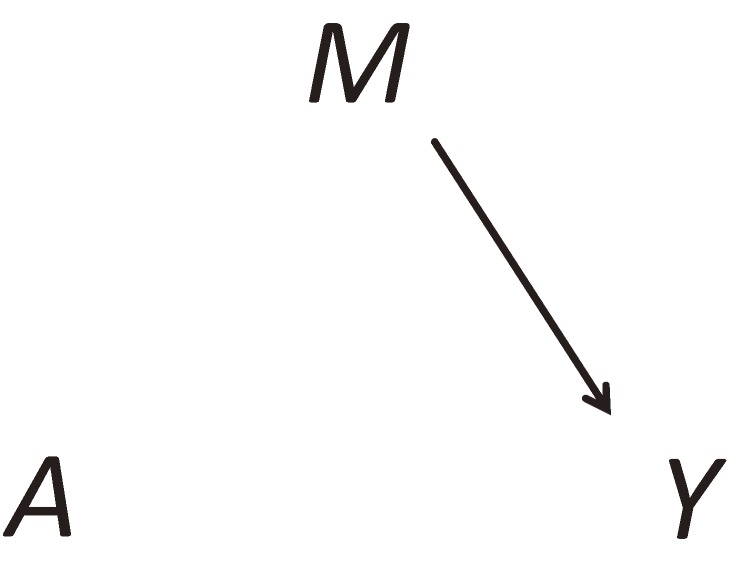
DAG that does not properly illustrate the underlying causal structures. We let *A* denote a binary exposure (1 = received heart transplant, 0 = did not receive heart transplant) and *Y* denote a binary outcome (1 = deceased, 0 = did not decease) and *M* denote a binary covariate (1 = men, 0 = women). DAG, directed acyclic graph.

## APPENDIX

As explained in Section 2-2 of the main text, an arrow from *P* to *Q* in a DAG indicates that *P* has a causal effect on *Q* of at least one individual in the population. In this Appendix, we illustrate this point following an example in Fine Point 6.2 of *Causal Inference: What If*.^5^ We let *A* denote a binary exposure (1 = received heart transplant, 0 = did not receive heart transplant) and *Y* denote a binary outcome (1 = deceased, 0 = did not decease) and *M* denote a binary covariate (1 = men, 0 = women). A half of the target population was men, and the other half was women. We consider a simple RCT, letting *p* denote a probability of exposure in the population, irrespective of sex. The assumptions about the two random variables *A* and *M* can be respectively described as:

$$A∼p_{A}(a)=\{\begin{array}{ll} p & \left(a=1\right)\\ 1-p & \left(a=0\right) \end{array}$$ (1)

and

$$M∼p_{M}(m)=\{\begin{array}{ll} 1/2 & \left(m=1\right)\\ 1/2 & \left(m=0\right) \end{array}$$ (2)

Consider a situation in which heart transplant *A* has a causal effect to increase the risk of death *Y* in every woman and has a causal effect to decrease the risk of death in every man. In other words, the response type of all the women is “causal” whereas that of all the men is “preventive”. Because the beneficial and harmful effects of *A* perfectly cancel out, the average causal effect in the target population is null. Despite the absence of average causal effect from *A* on *Y*, however, we need to draw an arrow from *A* to *Y* to make them d-connected because heart transplant *A* has a causal effect on the death *Y* of at least one (actually, all of the) individuals in the population (Figure 4). Below, we discuss this point by highlighting the difference between issues of causal structures and statistical (in)dependencies. Note that *M* is referred to as a direct effect modifier of *A* on *Y*.^70^

First, we discuss the relationship between *A*, *M*, and *Y* from the perspective of causal structures. Recall that a DAG represents a nonparametric structural equation model, and in Figure 4, we consider the following structural equations:

$$\{\begin{array}{l} A=f_{A}(\varepsilon_{A})\\ M=f_{M}(\varepsilon_{M})\\ Y=f_{Y}(A,M,\varepsilon_{Y}) \end{array}$$ (3)

where *f*(·) is an arbitrary function and ε represents all causes or random components of the corresponding variable not represented in the DAG. Note that these random components are independent of each other. Suppose that the nonparametric structural equation models in this example are parametrized as:

$$\{\begin{array}{l} A=f_{A}(\varepsilon_{A})\\ M=f_{M}(\varepsilon_{M})\\ Y=f_{Y}(A,M)=A(1-M)+(1-A)M \end{array}$$ (4)

Note that we dropped off ε*_Y_* from Equation 4 because we consider an example in which *Y* is fully determined by *A* and *M*. Then, among men (ie, *M* = 1), the structural equation for *Y* in Equation 4 becomes

$$Y=f_{Y}(A,1)=1-A$$ (5)

which shows that the response type of all the men is “preventive”. By contrast, among women (ie, *M* = 0), the structural equation for *Y* in Equation 4 becomes

$$Y=f_{Y}(A,0)=A$$ (6)

which shows that the response type of all the women is “causal”. Note that Equations 5 and 6 show that *Y* is causally determined by *A* conditional on *M*. This is graphically illustrated as the arrow from *A* to *Y* in the DAG with a box around *M* (Figure 5). Thus, *A* and *Y* are conditionally d-connected.

Next, we discuss the relationship between *A*, *M*, and *Y* from the perspective of statistical (in)dependencies. From Equations 1, 2, and 4, the joint distribution of (*A*, *M*, *Y*) is described as:

$$\begin{array}{l} \left(A,M,Y)∼p_{AMY}(a,m,y\right)\\ \;=\{\begin{array}{ll} 1/2\cdot p & \left(a,m,y)=(1,1,0),(1,0,1\right)\\ 1/2\cdot (1-p) & \left(a,m,y)=(0,1,1),(0,0,0\right)\\ 0 & \text{otherwise} \end{array} \end{array}$$ (7)

By marginalizing over *M*, the joint distribution of (*A*, *Y*) is given as:

$$(A,Y)∼p_{AY}(a,y)=\{\begin{array}{ll} 1/2\cdot p & \left(a,y)=(1,0),(1,1\right)\\ 1/2\cdot (1-p) & \left(a,y)=(0,1),(0,0\right) \end{array}$$ (8)

Further, by marginalizing over *A*, the marginal distribution of *Y* is given as:

$$Y∼p_{Y}(y)=\{\begin{array}{ll} 1/2 & \left(y=1\right)\\ 1/2 & \left(y=0\right) \end{array}$$ (9)

which shows that the probability of death in the total population is 50% because the numbers of men and women are equal in this example. From Equations 1, 8, and 9, we obtain

$$\forall (a,y),\;p_{AY}(a,y)=p_{A}(a)p_{Y}(y)$$ (10)

which clearly shows that *A* and *Y* are statistically independent when we do not condition on *M*. When we condition on *M*, the conditional distribution of (*A*, *Y*)|*_M_* is described as:

$$\begin{array}{ll} \left(A,Y){|}_{M=1}∼p_{AY|M}(a,y|m=1\right) & =\frac{p_{AMY}(a,m=1,y)}{p_{M}(m=1)}\\ & =\{\begin{array}{ll} p & \left(a,y)=(1,0\right)\\ 1-p & \left(a,y)=(0,1\right)\\ 0 & \text{otherwise} \end{array}\\ \left(A,Y){|}_{M=0}∼p_{AY|M}(a,y|m=0\right) & =\frac{p_{AMY}(a,m=0,y)}{p_{M}(m=0)}\\ & =\{\begin{array}{ll} p & \left(a,y)=(1,1\right)\\ 1-p & \left(a,y)=(0,0\right)\\ 0 & \text{otherwise} \end{array} \end{array}$$ (11)

Further, the conditional distributions of *A*|*_M_* and *Y*|*_M_* are respectively given as:

$$\begin{array}{l} A|{}_{M=1}∼p_{A|M}(a|m=1)=\{\begin{array}{ll} p & \left(a=1\right)\\ 1-p & \left(a=0\right) \end{array}\\ A{|}_{M=0}∼p_{A|M}(a|m=0)=\{\begin{array}{ll} p & \left(a=1\right)\\ 1-p & \left(a=0\right) \end{array} \end{array}$$ (12)

and

$$\begin{array}{l} Y{|}_{M=1}∼p_{Y|M}(y|m=1)=\{\begin{array}{ll} 1-p & \left(y=1\right)\\ p & \left(y=0\right) \end{array}\\ Y{|}_{M=0}∼p_{Y|M}(y|m=0)=\{\begin{array}{ll} p & \left(y=1\right)\\ 1-p & \left(y=0\right) \end{array} \end{array}$$ (13)

From Equations 11 to 13, we obtain

$$\begin{array}{l} p_{AY|M}(a=1,y=0|m=1)\\ \;=p\neq p\cdot p=p_{A|M}(a=1|m=1)\cdot p_{Y|M}(y=0|m=1)\\ p_{AY|M}(a=1,y=1|m=0)\\ \;=p\neq p\cdot p=p_{A|M}(a=1|m=0)\cdot p_{Y|M}(y=1|m=0) \end{array}$$ (14)

which shows that *A* and *Y* are statistically dependent conditional on *M*.

Then, what if one draws an arrow from *A* to *Y* based on the statistical (in)dependencies between them? Because *A* and *Y* are statistically independent without conditioning on *M*, one would not draw an arrow from *A* to *Y* in the DAG without a box around *M* (ie, d-separation of *A* and *Y*) as shown in Figure 6. Note that one (implicitly) employ an assumption of *faithfulness* here. By contrast, because *A* and *Y* are statistically dependent conditional on *M*, one would draw an arrow from *A* to *Y* in the DAG with a box around *M* (ie, conditional d-connection of *A* and *Y*) as shown in Figure 5, which is based on the rule of *compatibility*. Therefore, if one draws an arrow from *A* to *Y* based on the statistical (in)dependencies, the presence of the arrow depends on the presence of a “box” around *M*. In other words, the presence of the arrow from *A* to *Y* becomes “unstable”.

To summarize, although *A* and *Y* are statistically independent without conditioning on *M*, we need to draw an arrow from *A* to *Y* irrespective of whether we draw a box around *M* or not. This is because *Y* is causally determined by *A*. Thus, Figure 4 and Figure 5 properly illustrate the underlying causal structures, but Figure 6 does not. Under the rule of compatibility, d-connection is a necessary but not a sufficient condition for statistical dependence. The above discussion exemplifies that an arrow in DAGs represents the presence of individual causal effect in the target population.
